# Supplementary material for: Growth differentiation factor 1-induced tumour plasticity provides a therapeutic window for immunotherapy in hepatocellular carcinoma
Source: Nat Commun. 2021 Dec 8;12:7142. doi: 10.1038/s41467-021-27525-9 (PMC8654996; doi:10.1038/s41467-021-27525-9)
Supplement: Supplementary file 1 — Supplementary imformation [file 41467_2021_27525_MOESM1_ESM.pdf]

Supplementary information

Growth Differentiation Factor 1-Induced Tumour Plasticity Provides A Therapeutic Window for Immunotherapy in Hepatocellular Carcinoma

List of contents

Supplementary Fig.1: Expression of GDF1 in HCC tissues, cell lines and the *in vitro* functions

Supplementary Fig.2: The regulation of GDF1 on HCC lineage differentiation markers and their clinical association in HCC patients

Supplementary Fig.3: GDF1 activated through ALK7 for the oncogenic phenotypes

Supplementary Fig.4: GDF1 activated a broad panel of cancer testis antigens (CTAs) in HCC

Supplementary Fig.5: GDF1 suppressed the expression of epigenetic regulator LSD1

Supplementary Fig.6: The specified classifiers to divided HCC patients from the TCGA-LIHC project into 3 immunophenotypes

Supplementary Fig.7: GDF1-induced tumour lineage plasticity might sensitize HCC patients to anti-PD1 therapy

Supplementary Fig.8: Combination of LSD1 inhibitor and anti-PD1 antibody might provide novel therapeutic strategy for HCC patients

Supplementary Fig.9: QC metrics of RNA sequencing experiment performed in PLC-8024-CTR and PLC-8024-GDF1 cells

Supplementary Table 1: The association between GDF1 expression and clinical pathological features in TMA containing 196 HCC Patients

Supplementary Table 2: Univariate and multivariate Cox regression analysis of clinical pathological features associated with overall survival

Supplementary Table 3: Clinical characteristics of the patients in HKU cohort, TCGA cohort

Supplementary Table 4: Sequences of primers used in qPCR

Supplementary Table 5: Target sequences of shRNA and gRNA

Supplementary Figures

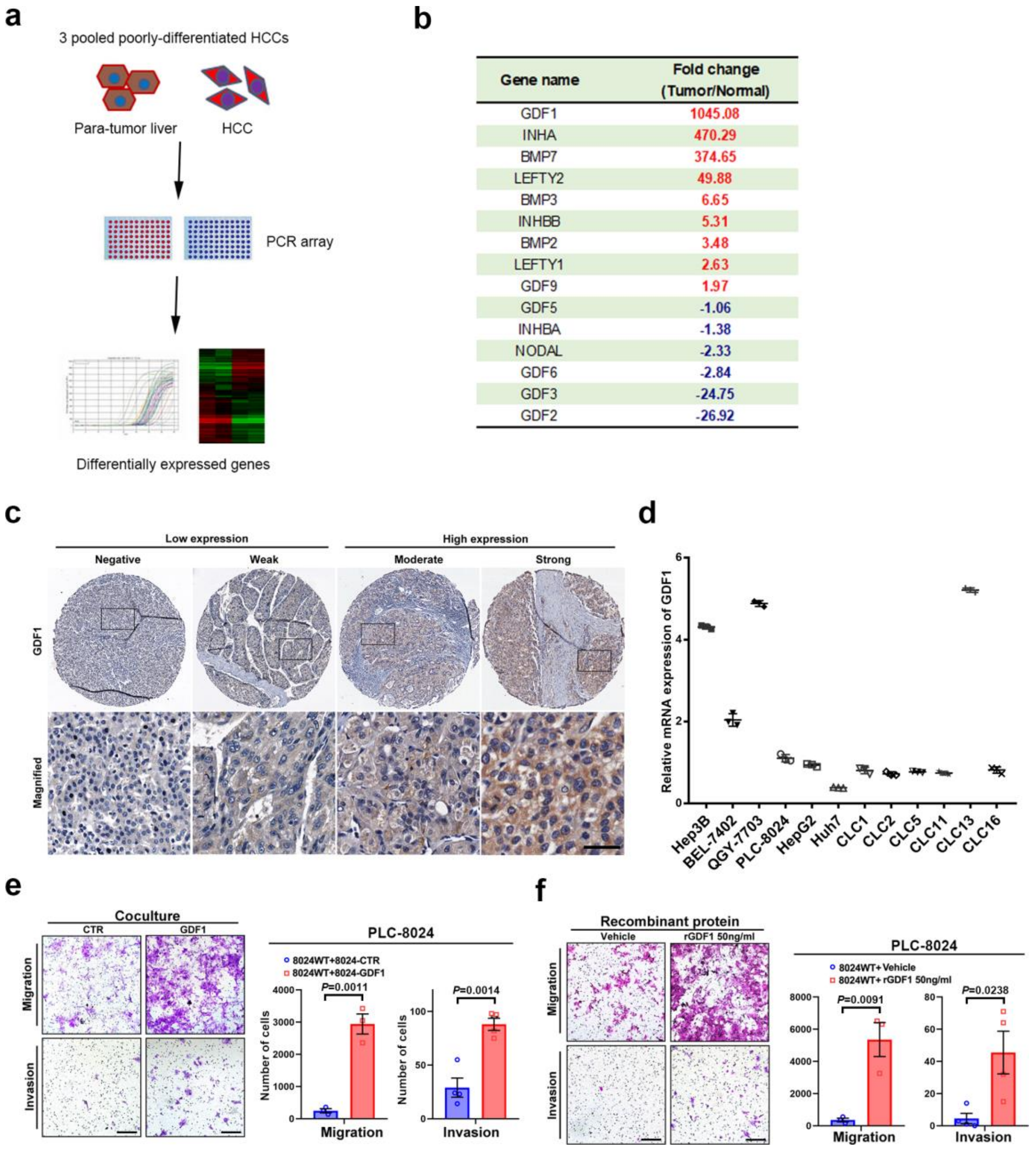

Supplementary Fig.1: Expression of GDF1 in HCC tissues, cell lines and the *in vitro* functions

a PCR array containing detection probes for genes encoding 375 secreting chemokines or cytokines, were performed in 3 pooled HCC tissues with poor differentiation and their paired non-tumour liver tissues. **b** The most differentially expressed genes were enriched in the TGF-β superfamily members. **c** Representative images of GDF1 immunohistochemical staining at negative, low, moderate and high staining intensities in TMA containing 196 liver tumour tissues from HCC patients. Scale bar, 50μm, n= 3 independent experiments. **d** GDF1 expressions in 10 HCC cell lines were tested by qPCR (Data are presented as mean values SD, data are the representative of three independent experiments). **e, f** Cell

migration and invasion abilities were detected in wildtype PLC-8024 cells cocultured with PLC-8024-GDF1 cells (**e**), or addition of recombinant GDF1 protein (**f**) at concentration of 50 ng/mL (Two-tailed independent Student s *t* test, data are presented as mean values SEM, n= 3-4 independent experiments). Source data are provided as a Source Data file.

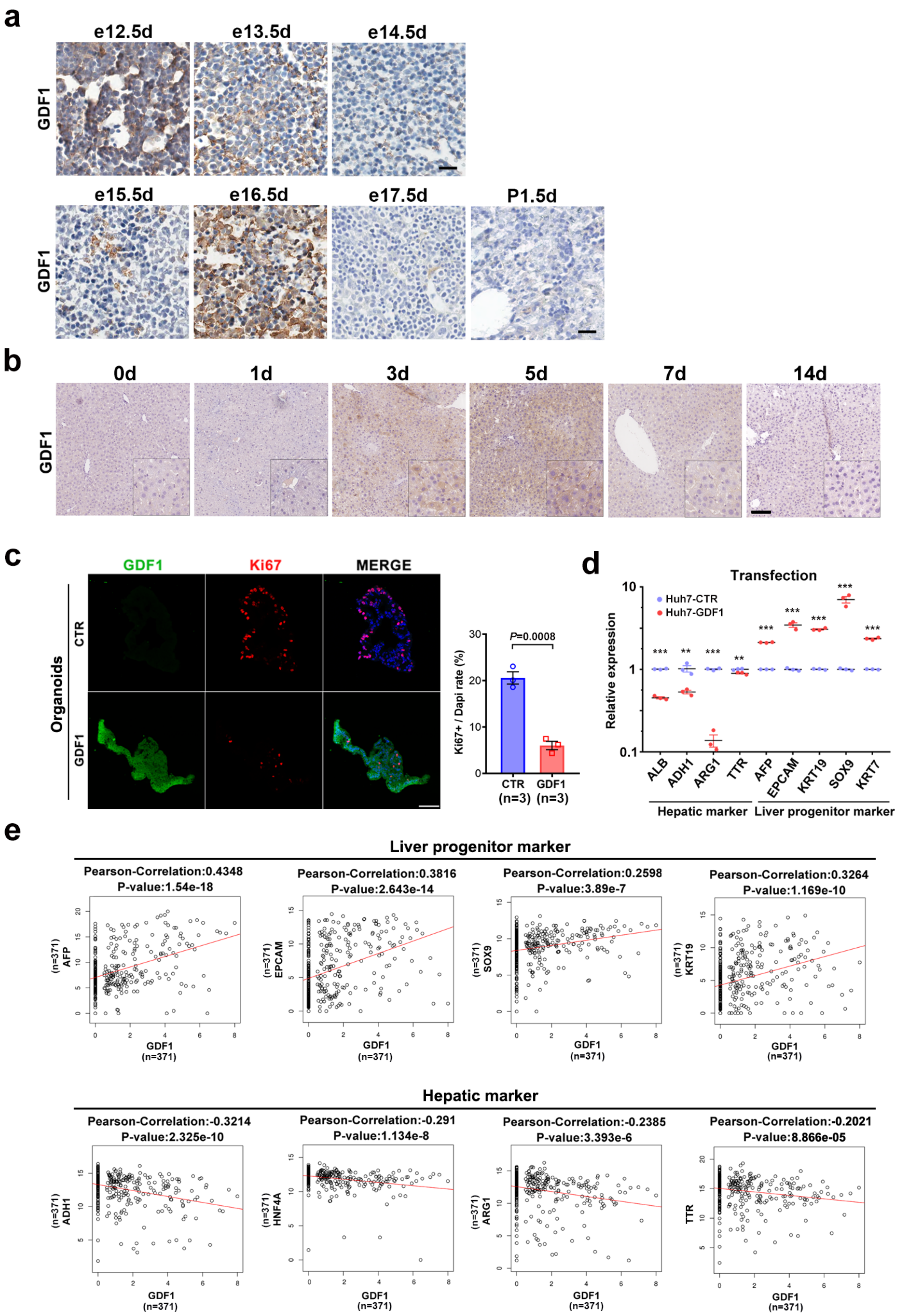

Supplementary Fig.2: The regulation of GDF1 on HCC lineage differentiation markers and their clinical association in HCC patients

a IHC staining of GDF1 in fetal mice liver. Scale bar, 20μm, n= 3 independent experiments. **b** IHC staining of GDF1 in regenerating mice liver after partial hepatectomy. Scale bar, 100μm, n= 3 independent experiments. **c** Immunofluorescent costaining of GDF1 with Ki67 in primary mice hepatocytes, which were cultured in an organoid model, and subjected to lenti-virus-mediated transfection of GDF1 or control vector. Scale bar, 50μm. (Two-tailed independent Student's *t* test, data are presented as mean values ± SEM, n= 3 independent experiments). **d** Representative hepatic markers (ALB, ARG1, TTR, HNF4A) and liver progenitor markers (AFP, EPCAM, KRT19, SOX9, KRT7) were examined by qPCR in Huh7 cells stably transfected with or without GDF1 (**, ***P***<0.01, ***, ***P***<0.001, two-tailed independent Student's *t* test, data are presented as mean values ± SD, data are the representative of three independent experiments). **e** Correlation analyses between the expressions of GDF1, liver progenitor markers and hepatic markers in HCC by using Linkedomics web server based on the TCGA database (n=371). Source data are provided as a Source Data file.

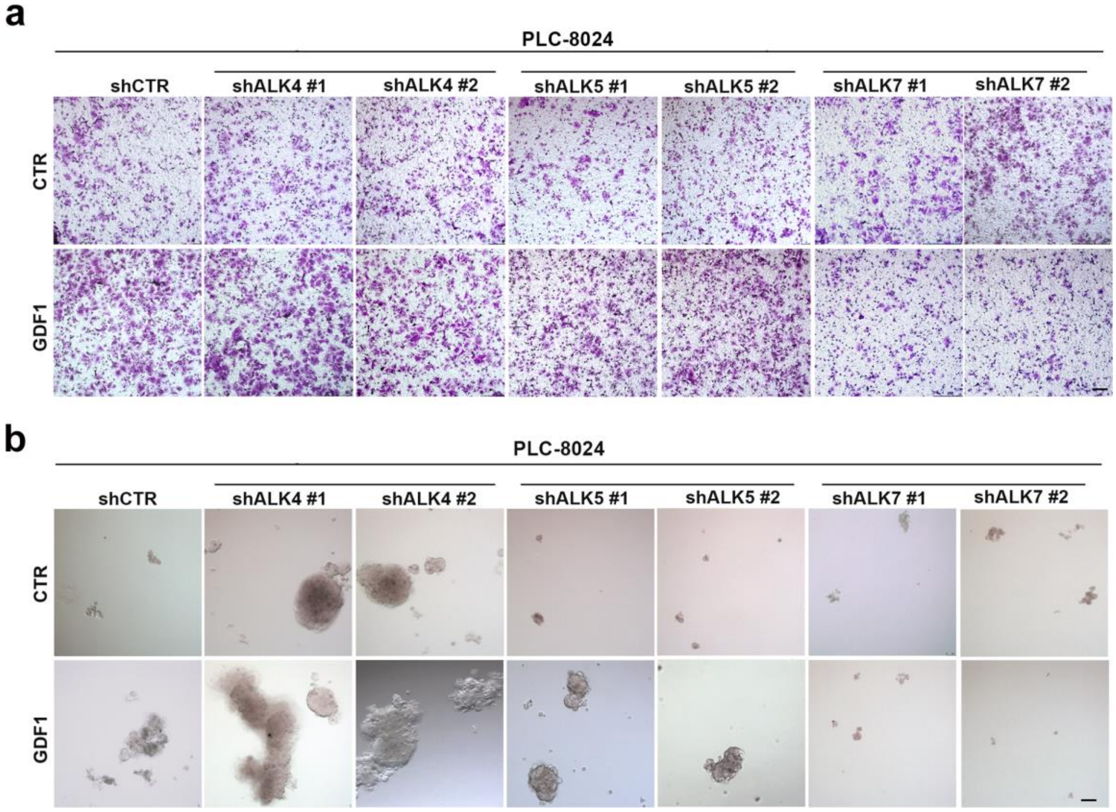

Supplementary Fig.3: GDF1 activated through ALK7 for the oncogenic phenotypes

a PLC-8024 cells transfected with or without GDF1 were transduced with lentivirus-mediated shRNAs specifically targeting different ALK4, ALK5, ALK7, respectively. Cell migration assays were performed in different targeting groups. Scale bars represent 100 µm. **b** Sphere formation assays were performed in different targeting groups. Scale bars represent 100 µm. All data are the representative of three independent experiments.

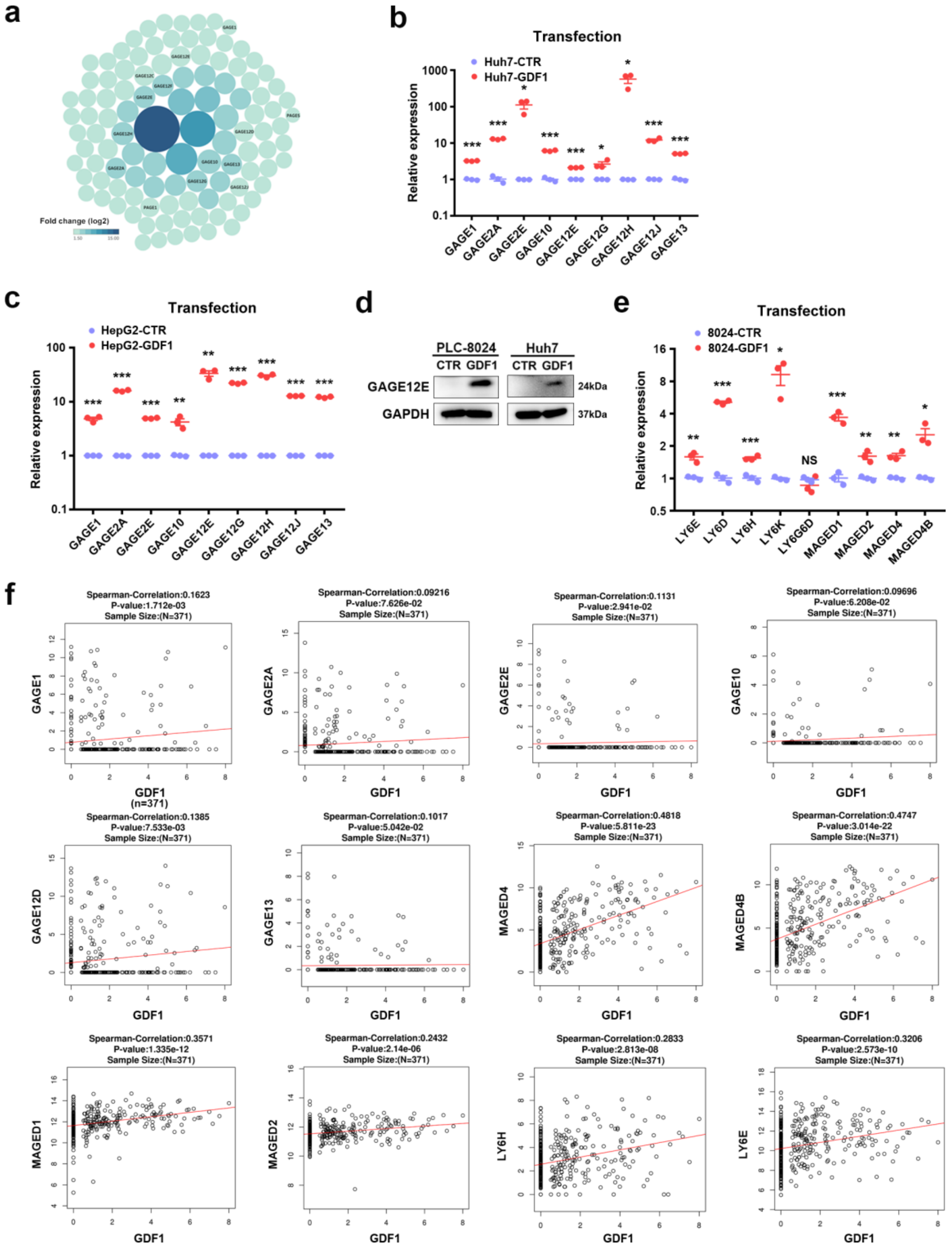

Supplementary Fig.4: GDF1 activated a broad panel of cancer testis antigens (CTAs) in HCC

a RNA-seq was performed in PLC-8024-CTR and PLC-8024-GDF1 cells. Bubble chart indicated that the most differentially expressed genes were enriched in CTAs including GAGE family members. **b** The relative expression of representative CTAs was detected by qPCR in Huh7-CTR and Huh7-GDF1 cells (*, ***P***<0.05, ***, ***P***<0.001, two-tailed independent Student's *t* test, data are presented as mean values ± SD, data are the representative of three independent experiments). **c** The relative expression of

representative CTAs was detected by qPCR in HepG2-CTR and HepG2-GDF1 cells (**, ***P***<0.01, ***, ***P***<0.001, two-tailed independent Student's *t* test, data are presented as mean values ± SD, data are the representative of three independent experiments). **d** The expression of GAGE12E at the protein level was detected by Western Blot in PLC-8024 and Huh7 cells transfected with or without GDF1 (Data are the representative of three independent experiments). **e** The relative expression of other CTAs including MAGE and LY6 family members was detected by qPCR in PLC-8024-CTR and PLC-8024-GDF1 cells (*, ***P***<0.05, **, ***P***<0.01, ***, ***P***<0.001, NS, not significant, two-tailed independent Student's *t* test, data are presented as mean values ± SD, data are the representative of three independent experiments). **f** Correlation analyses between the expressions of GDF1, GAGE family members, MAGE and LY6 family members in HCC by using Linkedomics web server based on the TCGA database (n=371). Source data are provided as a Source Data file.

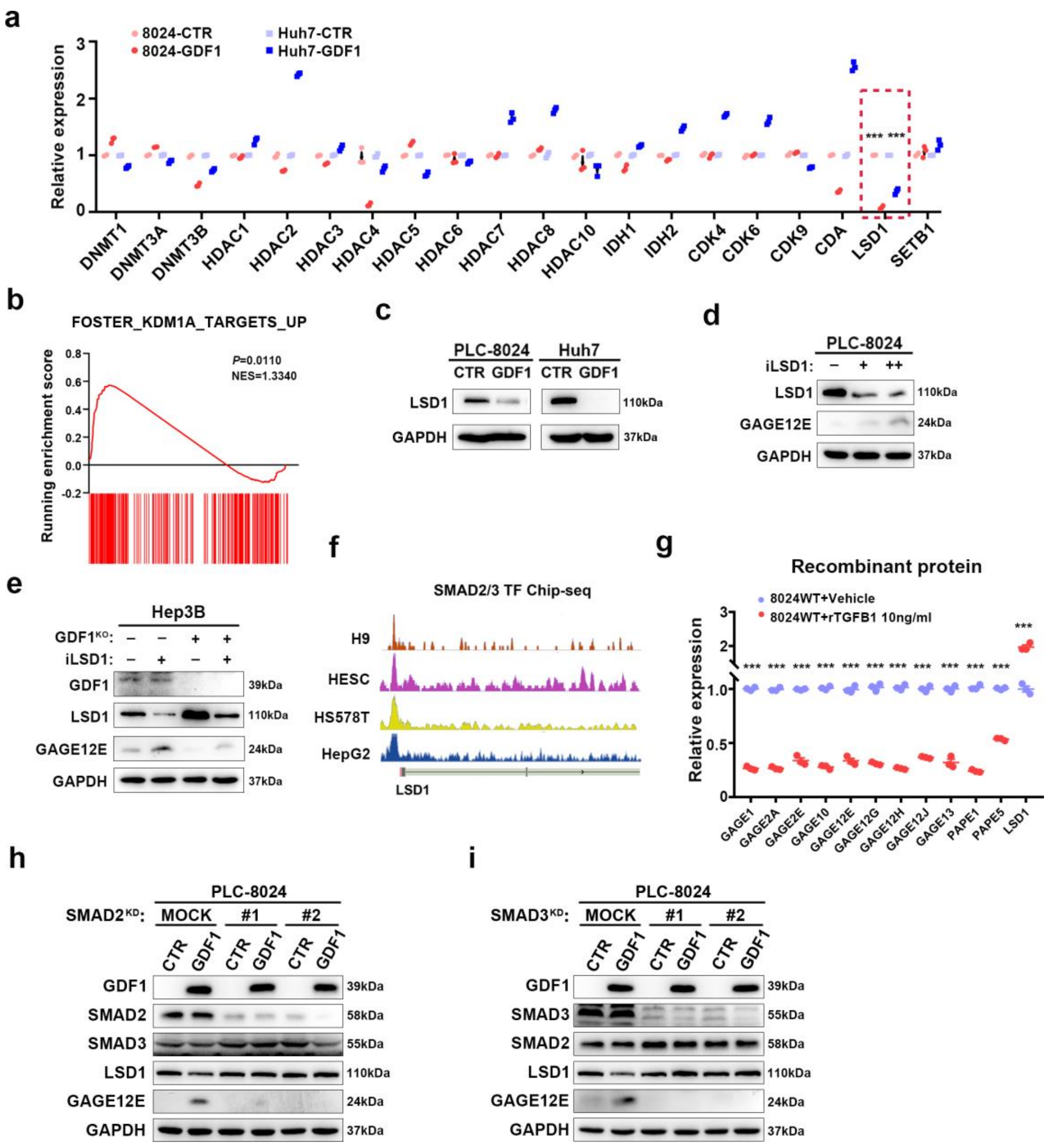

Supplementary Fig.5: GDF1 suppressed the expression of epigenetic regulator LSD1

a The relative expression of a panel of epigenetic modulators was detected by qPCR in PLC-8024 and Huh7 cells transfected with or without GDF1 (***, ***P***<0.001, two-tailed independent Student's *t* test, data are presented as mean values ± SD, data are the representative of three independent experiments). **b** GSEA of LSD1 target genes signature in PLC-8024-GDF1 cells versus control cells. NES, normalized enrichment score. **c** The expression of LSD1 at the protein level was detected by Western Blot in PLC-8024 and Huh7 cells transfected with or without GDF1 (Data are the representative of three independent experiments). **d** PLC-8024 cells were treated with LSD1 inhibitor at 5 μM or 10 μM for 72 hours. The expression of GAGE12E and LSD1 at the protein level was examined by western blot. iLSD1, GSK-LSD1. +, 5 μM. ++,10 μM (Data are the representative of three independent experiments). **e** Hep3B-

CTR and Hep3B GDF1^{KO} cells were treated with LSD1 inhibitor at 5 μM or 10 μM for 72 hours. The expression of GAGE12E and LSD1 at the protein level was examined by western blot. iLSD1, GSK-LSD1. +, 5 μM. ++,10 μM (Data are the representative of three independent experiments). **f** Data from the Encyclopedia of DNA Elements database indicated the occupation of SMAD2/3 on the promoter region of LSD1 in several cell lines. **g** The relative expression of representative CTAs was detected by qPCR in PLC-8024 cells treated with rTGFB1 at 10 ng/mL or vehicle control for 15 days (***, ***P***<0.001, two-tailed independent Student's *t* test, data are presented as mean values ± SD, data are the representative of three independent experiments). **h, i** The expression of LSD1 and GAGE12E at the protein level was detected by Western Blot in PLC-8024-CTR and PLC-8024-GDF1 cells transduced with lentivirus-mediated shRNAs specifically targeting SMAD2 (**h**) or SMAD3 (**i**) (Data are the representative of three independent experiments). Source data are provided as a Source Data file.

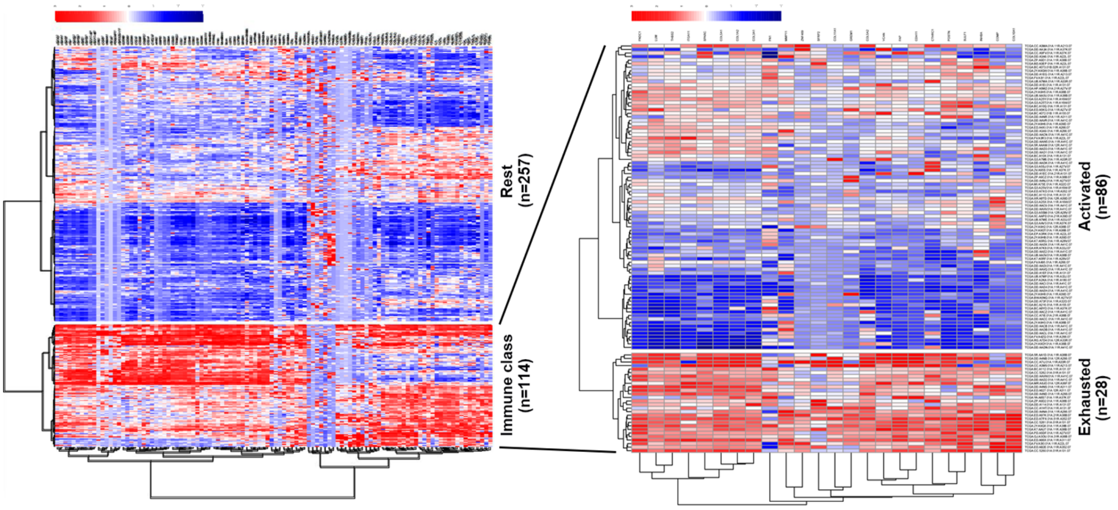

Supplementary Fig.6: The specified classifiers to divided HCC patients from the TCGA-LIHC project into 3 immunophenotypes

HCC patients from the TCGA-LIHC project were divided into 3 subgroups (the activated immune group, exhausted immune group, and remaining group) according to the specified classifiers.

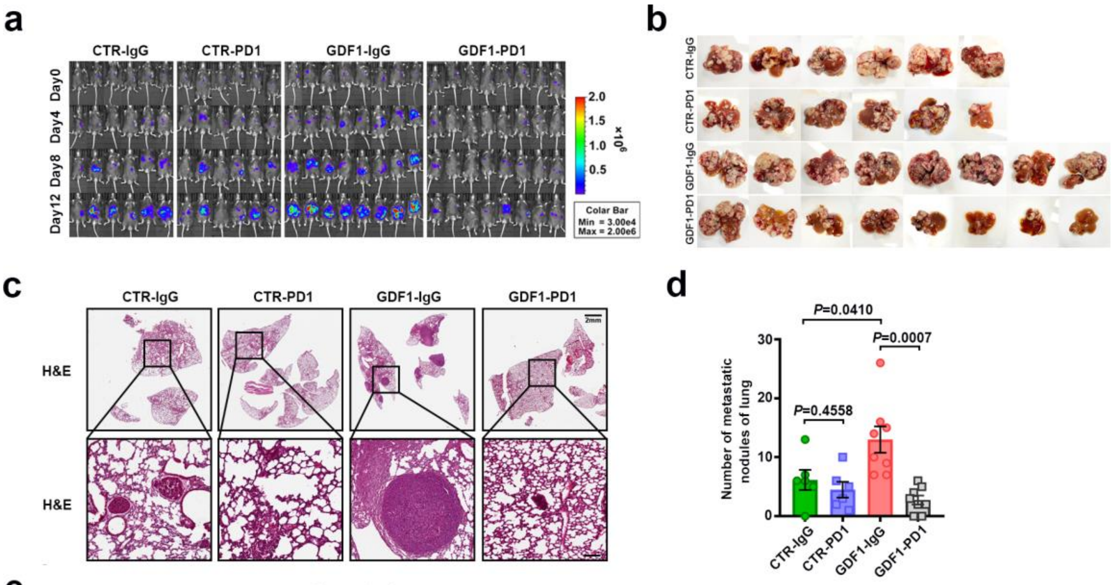

e

Correlations

					CD8 expression	GDF1 expression
Spearman's rho	CD8expression	Correlation Coefficient			1.000	.546**
		Sig. (2-tailed)			.	.000
		N			196	196
		Bootstrap[b]	Bias		.000	.000
			Std. Error		.000	.054
			95% Confidence Interval	Lower	1.000	.431
				Upper	1.000	.642
	GDF1expression	Correlation Coefficient			.546**	1.000
		Sig. (2-tailed)			.000	.
		N			196	196
		Bootstrap[b]	Bias		-.001	.000
			Std. Error		.054	.000
			95% Confidence Interval	Lower	.431	1.000
				Upper	.642	1.000

**. Correlation is significant at the 0.01 level (2-tailed).

b. Unless otherwise noted, bootstrap results are based on 1000 bootstrap samples

f

		NO. expression of CD8(%)					
		-	+	++	+++	Total	*P*-value
GDF1	-	24 (77.4%)	5 (16.1%)	1 (3.2%)	1 (3.2%)	31 (100%)	
	+	17 (44.7%)	13 (34.2%)	8 (21.1%)	0 (0.0%)	38 (100%)	
	++	16 (20.0%)	38 (47.5%)	20 (25.0%)	6 (7.5%)	80 (100%)	
	+++	1 (2.1%)	17 (36.2%)	15 (31.9%)	14 (29.8%)	47 (100%)	
	Total	58 (29.6%)	73 (37.2%)	44 (22.4%)	21 (10.7%)	196 (100%)	$P < 0.0001$

Supplementary Fig.7: GDF1-induced tumour lineage plasticity might sensitize HCC patients to anti-PD1 therapy

a Representative bioluminescence images of mice bearing orthotopic Hepa1-6 tumours after anti-PD1 antibody or rat IgG2a control therapy. **b** Representative photographs of liver tumours after anti-PD1 antibody or rat IgG2a control treatment. **c** HE staining of representative metastatic lung nodules after anti-PD1 antibody or rat IgG2a control treatment. Scale bar, 200μm. **d** The number of metastatic lung nodules in mice bearing Hepa1-6 tumours transfected with mGDF1 after anti-PD1 antibody or rat IgG2a control therapy (Two-tailed independent Student's *t* test, data are presented as mean values ± SEM, CTR-IgG: n = 6, CTR-PD1: n = 6, GDF1-IgG: n = 8, GDF1-PD1: n = 8). **e** Spearsman correlation analyses (Two-tailed) was used for GDF1 and CD8 expression in HCC patients from the TMA. **f** Crosstab of

GDF1 and CD8 consecutive staining in HCC patients from the TMA. ***P***<0.0001, Two–sided $\chi 2$ test. Source data are provided as a Source Data file.

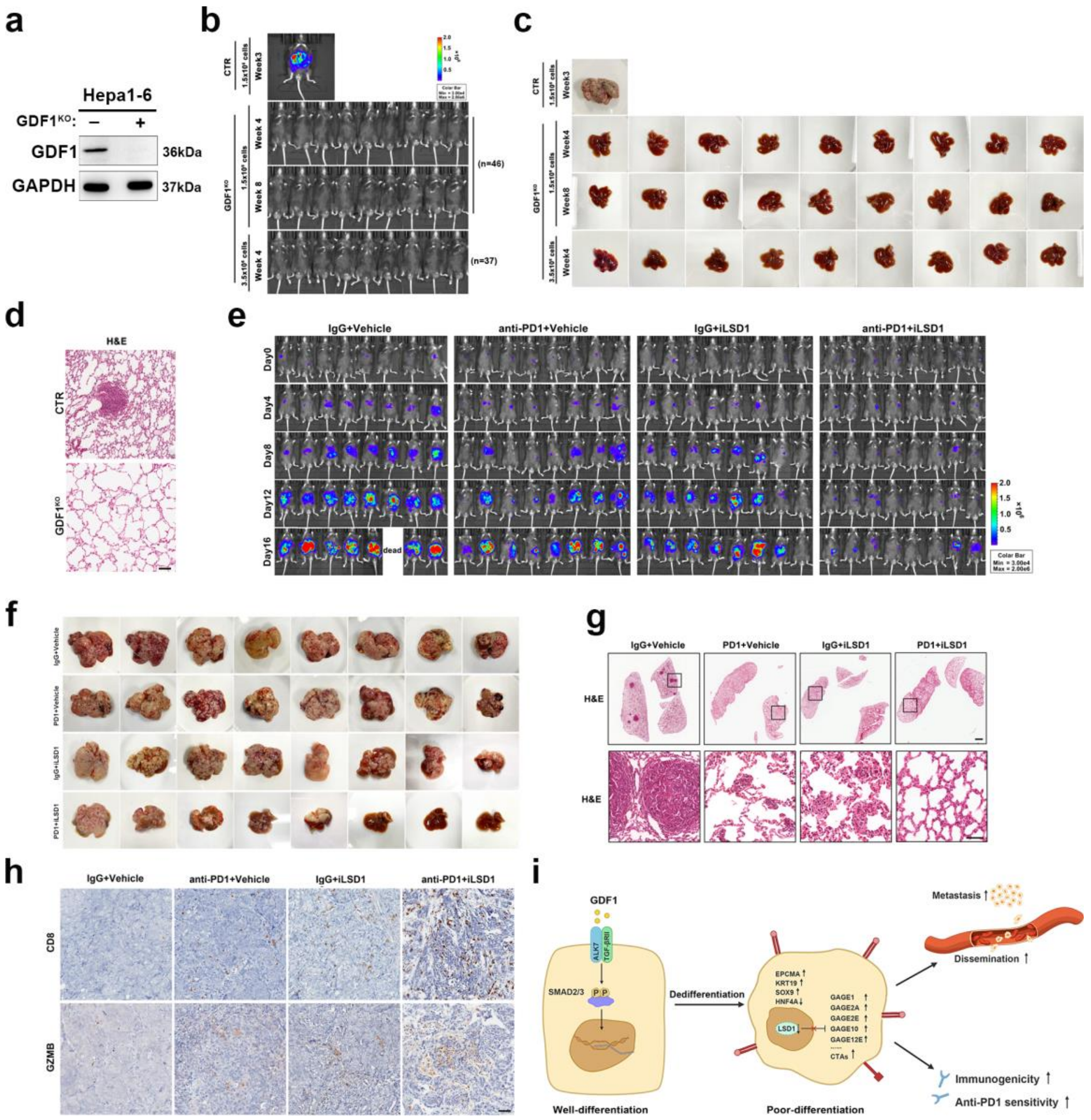

Supplementary Fig.8: Combination of LSD1 inhibitor and anti-PD1 antibody might provide novel therapeutic strategy for HCC patients

a The expression of GDF1 at the protein level was detected by Western Blot in Hepa1-6 CTR or Hepa1-6 GDF1^{KO} cells (Data are the representative of three independent experiments). **b** To establish a luciferase-labelled metastatic HCC model, different number of Hepa1-6-CTR or Hepa1-6-GDF1^{KO} cells stably expressing firefly luciferase were intrasplenically injected into C57BL/6 mice. Representative bioluminescence images of mice bearing metastatic Hepa1-6 CTR or Hepa1-6 GDF1^{KO} tumours on different days post tumour inoculation. Top panel: Hepa1-6 CTR, 1.5×10^{6} cells/mouse, 3 weeks later; Middle panel: Hepa1-6-GDF1^{KO}, 1.5×10^{6} cells/mouse, 4 weeks and 8weeks later (n=46); Bottom panel: Hepa1-6-GDF1^{KO}, 3.5×10^{6} cells/mouse, 4 weeks later (n=37). **c** Representative photographs of liver tumours after inoculating different number of Hepa1-6-CTR or Hepa1-6-GDF1^{KO} cells. Top panel:

Hepa1-6 CTR, 1.5×10^6 cells/mouse, 3 weeks later; Middle panel: Hepa1-6-GDF1^{KO}, 1.5×10^6 cells/mouse, 4 weeks and 8 weeks later (n = 46, 10 mice were euthanized to collect tumours at 4 weeks, while the rest continued to evaluate tumour formation and metastasis until 8 weeks post tumour inoculation); Bottom panel: Hepa1-6-GDF1^{KO}, 3.5×10^6 cells/mouse, 4 Weeks post tumour inoculation (n =37). **d** HE staining were performed in metastatic lung tumour nodules (Data are the representative of ten independent experiments). **e** Representative bioluminescence images of mice bearing metastatic Hepa1-6-CTR tumours after combination treatment of LSD1 inhibitor with anti-PD1 antibody. **f** Representative photographs of liver tumours after combination treatment of LSD1 inhibitor with anti-PD1 antibody. **g** HE staining of representative metastatic lung nodules after combination treatment of LSD1 inhibitor with anti-PD1 antibody (n= 8 mice per group). **h** IHC staining of CD8 and GZMB in mouse orthotopic Hepa1-6-CTR tumours after combination treatment of LSD1 inhibitor with anti-PD1 antibody. Scale bars represent 50 µm, n= 5 independent experiments. **i** Schematic figure summarizing the functions of GDF1 in HCC. Source data are provided as a Source Data file.

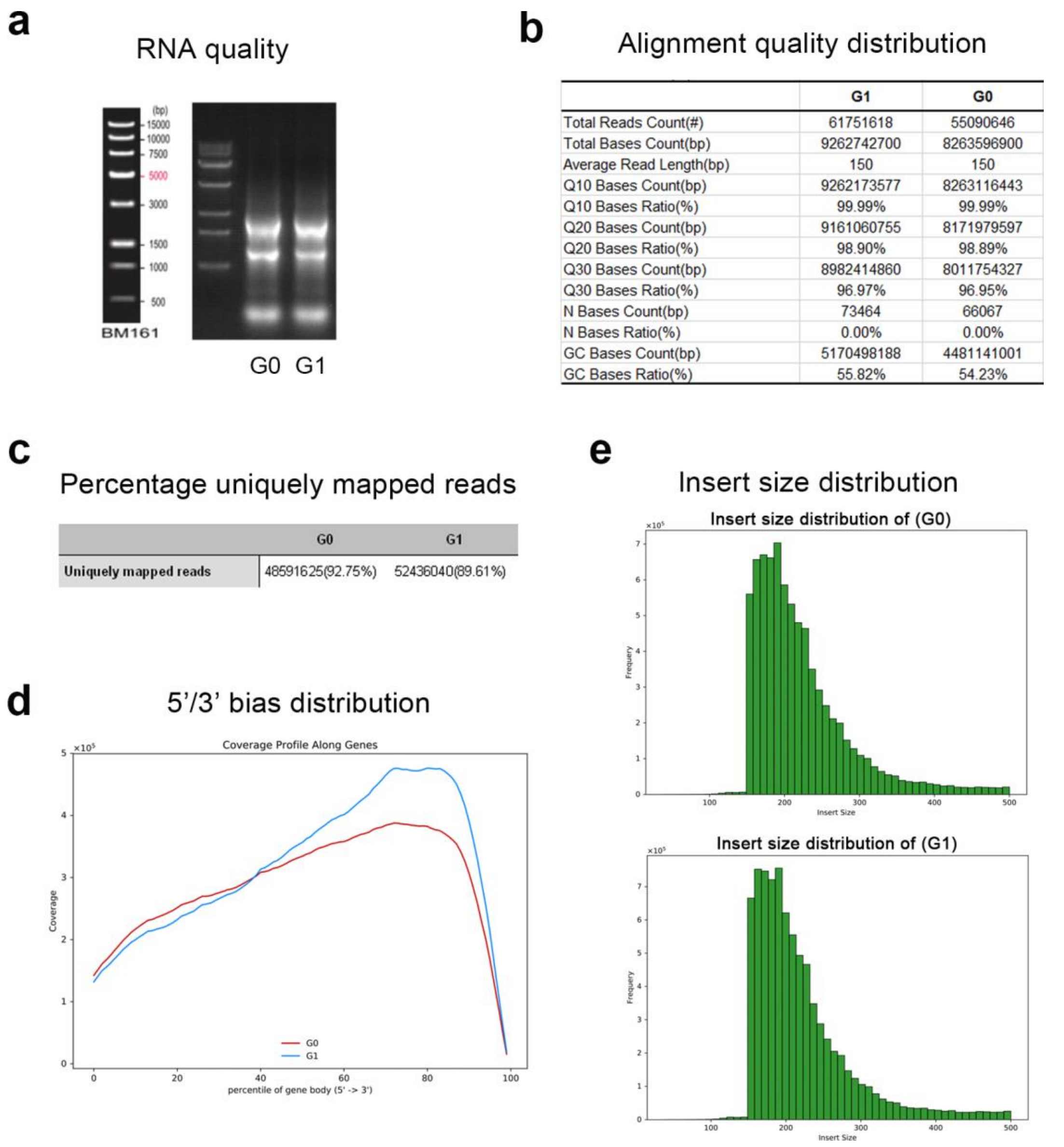

Supplementary Fig.9: QC metrics of RNA sequencing experiment performed in PLC-8024-CTR and PLC-8024-GDF1 cells

a Agarose gel electrophoresis of RNA used in RNA sequencing experiment. **b** Alignment quality distribution of RNA sequencing experiment. **c** Percentage uniquely mapped reads of RNA sequencing experiment. **d** 5'/3' bias distribution of RNA sequencing experiment. **e** Insert size distribution of RNA sequencing experiment. G0, PLC-8024-CTR. G1, PLC-8024-GDF1.

Supplementary Table 1 The association between GDF1 expression and clinical pathological features in TMA containing 196 HCC Patients.

Clinical pathological features	Total	GDF1 expression		*P**
		Low	High	
**Gender**				
Male	166	57	109	0.550
Female	30	12	18	
**Age**				
≤ 50	107	41	66	0.317
> 50	89	28	61	
**Preoperative AFP**				
< 400 ng/ml	107	43	64	0.109
≥400 ng/ml	89	26	63	
**Preoperative ALT**				
≤ 40 U/L	101	41	60	0.103
> 40 U/L	95	28	67	
**Preoperative AST**				
≤ 40 U/L	116	47	69	0.061
> 40 U/L	80	22	58	
**Preoperative ALB**				
< 35 g/L	6	2	4	0.922
≥ 35 g/L	190	67	123	
**HBsAg**				
Negative	31	14	17	0.206
Positive	165	55	110	
**Tumor Stage**				
Ⅰ & Ⅱ	141	56	85	**0.034**
Ⅲ&Ⅳ	55	13	42	
**Tumor Size**				

≤ 5 cm	75	22	53	0.175
> 5 cm	121	47	74	
**Multiple Tumor**				
No	153	57	96	0.257
Yes	43	12	31	
**Tumor Embolus**				
No	160	61	99	0.071
Yes	36	8	28	
**Adjacent Invasion**				
No	188	69	119	**0.033**
Yes	8	0	8	
**Tumor Relapse**				
No	70	34	36	**0.003**
Yes	126	35	91	
**Differentiation**				
Well	17	14	3	**0.000**
Moderate	98	46	52	
Poor	81	9	72	

*: Two–sided χ2 test.

Supplementary Table 2 Univariate and multivariate Cox regression analysis of clinical pathological features associated with overall survival.

**Clinical Pathological Features**	**Univariate Analysis**			**Multivariate Analysis**		
	HR	95% CI	P value	HR	95% CI	*P**value
**Age** (year)						
>50 vs. ≤50	1.346	0.912-1.985	0.134	NA	NA	NA
**Gender**						
Female vs. Male	0.617	0.382-0.998	**0.049**	0.648	0.391-1.074	0.092
**Tumor stage**						
III/IV vs. I/II	4.059	2.716-6.067	**0.000**	1.970	1.082-3.585	**0.026**
**HBV**						
HBV+ vs. HBV-	1.054	0.609-1.824	0.851	NA	NA	NA
**ALT** (U/L)						
>40 vs. ≤40	1.202	0.815-1.773	0.354	NA	NA	NA
**AST** (U/L)						
>40 vs. ≤40	2.003	1.357-2.958	**0.000**	1.197	0.795-1.803	0.389
**AFP** (ng/ml)						
≥400 vs. <400	1.690	1.145-2.495	**0.008**	1.094	0.706-1.693	0.688
**Multiple tumor**						
+ vs. -	2.154	1.408-3.296	**0.000**	0.985	0.553-1.755	0.959
**Tumor embolus**						
+ vs. -	2.132	1.364-3.333	**0.001**	1.140	0.688-1.888	0.612
**Tumor diameter** (cm)						
>5 vs. ≤5	2.030	1.318-3.126	**0.001**	1.112	0.662-1.866	0.688
**Tumor relapse**						
Yes vs. No	26.86	9.838-73.351	**0.000**	19.06	6.805-53.433	**0.000**
**Tumor differentiation**						
Poor vs. Well	1.966	1.332-2.903	**0.001**	1.440	0.920-2.253	0.110
**GDF1 expression**						
High vs. Low	1.923	1.269-2.915	**0.002**	2.382	1.399-4.055	**0.001**

*: Log-rank test.

Supplementary Table 3 Clinical characteristics of the patients in HKU cohort, TCGA cohort.

	HKU cohort	TCGA cohort
No. of patients	83	373
Gender, No. (%)		
Male	69 (83.1%)	252 (67.6%)
Female	14 (16.9%)	121 (32.4%)
Tumor Stage, No. (%)		
Ⅰ	36 (43.4%)	172 (46.1%)
Ⅱ	2 (2.4%)	87 (23.3%)
Ⅲ	20 (24.1%)	85 (22.8%)
Ⅳ	0 (0%)	5 (1.3%)
Tumor Grade, No. (%)		
G1	10 (12.0%)	55 (14.7%)
G2	41 (49.4%)	178 (47.7%)
G3&4	23 (27.7%)	135 (36.2%)
Cirrhosis, No. (%)		
No	25 (30.1%)	134 (35.9%)
Yes	53 (63.9%)	80 (21.4%)
Vascular invasion, No. (%)		
No	13 (15.7%)	207 (55.5%)
Yes	66 (79.5%)	110 (29.5%)
Recurrence, No. (%)		
No	46 (55.4%)	237 (63.5%)
Yes	35 (42.2%)	136 (36.5%)
HBsAg , No. (%)		
No	23 (27.7%)	232 (62.2%)
Yes	54 (65.1%)	141 (37.8%)
HCVAb , No. (%)		
No	76 (91.6%)	288 (77.2%)
Yes	1 (1.2%)	85 (22.8%)

Supplementary Table 4 Sequences of primers used in qPCR.

Gene	Forward (5’-3’)	Reverse (5’-3’)
18S	AACCCGTTGAACCCCATT	CCATCCAATCGGTAGTAGCG
GDF1	GGGTCGCCGGAAACATC	CAGCCGACAGGTCGAAGACG
AFP	CTTTGGGCTGCTCGCTATGA	GCATGTTGATTTAACAAGCTGCT
EPCAM	AATCGTCAATGCCAGTGTACTT	TCTCATCGCAGTCAGGATCATAA
KRT19	AACGGCGAGCTAGAGGTGA	GGATGGTCGTGTAGTAGTGGC
KRT7	TCCGCGAGGTCACCATTAAC	GCTCTGTCAACTCCGTCTCAT
SOX9	AGCGAACGCACATCAAGAC	CTGTAGGCGATCTGTTGGGG
HNF4A	CTGCCAATATCGCTACAAC	CATCCCTACGCTCCAGTA
ALB	TTTATGCCCCGGAACTCCTTT	AGTCTCTGTTTGGCAGACGAA
TTR	CATTTGCCTCTGGGAAAACCAG	AATGGGGAGATGCCAAGTGC
ARG1	TGGACAGACTAGGAATTGGCA	CCAGTCCGTCAACATCAAAACT
ALDH	AGTCATCCCACTCGCTATTCC	GTCCCCTGAGGATTGCTTACA
GAGE1	GATAGCCAGGAACAGGGTCAC	ACTGTGATTGCCCTTCACCTT
GAGE2A	GGGACAGGATGAGGGAGCA	CCATCAGGACCATCTTCACACTC
GAGE2E	GAGGAAGATCGACCTATCGGC	GGTGACCCTGTTCCTGGCTATC
GAGE10	CAGGAACAGGTTCACCCAAAG	CACCTCCTCTGGATTTGGCA
GAGE12E	AAGGGGAACCAGCAACTCAAT	GCAGATGCTCCCTCATCCTC
GAGE12G	GCCCGAGCAGTTCAGTGATG	CCTGTTCCTGGCTATGAGCTTC
GAGE12J	TATTGGCCTAGACCAAGACCCTA	TGACCCTGTTCCTGGCTATCA
GAGE12H	CTAGACCAAGGCGCTATGTACAG	TCCCTCATCCTCTCCCTTCTG
GAGE13	TTATTGGCCTAGACCAAGACGCTAC	TGACCCTGTTCCTGGCTATCA
PAGE1	CTATCGGCGTAGACCAA	CTCTTCATTTCGCAGGCACA
PAGE5	GCTGGAACGAGGGAGGAAG	TCAGTGGGCTGCTGGACAA
LY6D	TGAGGGGGAATCTGGTGAAG	ACAGGCTGGGGGCTAAGAT
LY6E	TAGTGCCGGCATTGGGAATC	CATCGGCCGCACTGAAATTG
LY6K	ACGGACGAGGGTGACAATAGAG	GCTTCGCAACCATGAAAAAAC

LY6H	CACCCTGACCACCAACTCCA	AACGAAGTCACAGGAGGAGGC
LY6G6D	CAAGTGGAGACAGAGTCGGTGG	AAGAGACAGGTCAGGGCGGTA
MAGED1	CCTGACGCTTGTAGAGCAGT	GAGGCCTCAGCAGGAGC
MAGED2	GGTGCGGCTGCAGAGAATTG	GCAAGAGCTGAGCCTTCCGTA
MAGED4	GCTGAGCTCTCATCTCCCTG	GTTCCCAGGGCTCATTCGAT
MAGED4B	CTTCTGCAGGAGAGGGCAAA	TTCCAGGGTGTACGTTGCTC
DNMT1	GACCCACGAAAGCCACCA	GACACACCTCACAGACGCCA
DNMT3A	CAGGATAGCCAAGTTCAGCAAA	GTGGACTGGGAAACCAAATACC
DNMT3B	TAAGTCGAAGGTGCGTCGTG	CGTCTTCGAGTCTTGTTCTCGTA
DNMT3L	AGTCAAGGCTAACCAGCGAAAT	CGCAGATCCCTCCCTCAAA
HDAC1	TTTGCTGCTCAACTATGGTCTCTAC	CGATGTCCGTCTGCTGCTTAT
HDAC2	GCTTGCCATCCTTGAATTACTAA	ATTGACAGCATAGTATTTGCCTTTT
HDAC3	TGCAAGGCTTCACCAAGAGTCT	GGATGCCAATCACAATGTCGTT
HDAC4	AAAAGCGTCCGTTGGATGTC	GGCAAGGATGGCGATGTCT
HDAC5	GCTGAGAATGGCTTTACTGGC	AGTTGGTGACAGTGACCGTGG
HDAC6	TATTTCTCCATCCACCGCTACG	ACCTGGTTCCAAGGCACATT
HDAC7	ATGACGCAGCAACTGATGAAC	GCGGATGGCATTGAGGTT
HDAC8	GCAATTAACTGGTCTGGAGGGT	GGAGGTGAAACTGAATGCGTCT
HDAC9	TGAGACGCAGACGCTTAGGC	CTGTCGCATTTGTTCTTTCAATAAT
HDAC10	ACTTCCACCCGAGTACCTTTCA	ACATCCCAGTCCACGACGAG
CDK4	CGTGAGGTGGCTTTACTGAGG	CTTGTCCAGATATGTCCTTAGGTCC
CDK6	CAGAAATGTTTCGTAGAAAGCCTCT	GGTCCTGGAAGTATGGGTGAGA
CDK9	CCAGAAGCGGAAGGTGAAG	CCAGAAGAAGTCGTGGTTGAGG
IDH1	TCAGTGGCGGTTCTGTGGTAG	CTTGGTGACTTGGTCGTTGGT
IDH2	AGAACACCATACTGAAAGCCTACG	CACACAAAGCCACCCGAAG
LSD1	TACCCGCTCCACGAGTCAA	GGATCAGCACGCCAACGA
SETDB1	TCGGGTGGTCGCCAAATA	GTGCCTTCCCACTCAGTCTTG
DOT1L	CCCCTCAACCTCAACTCCAT	GCCAAGCCACCACCATTTT

EZH2	GGACGGCTTCCCAATAACAG	ATTGAGGCTTCAGCACCACT
AICDA	TCAAAAATGTCCGCTGGGC	TTCCCTCGCAGAAAGTCGG
CDA	GAAGTCAGCCTACTGCCCCTAC	AGCGGGTAGCAGGCATTTT
m18S	AGGGGAGAGCGGGTAAGAGA	GGACAGGACTAGGCGGAACA
mGDF1	AACTAGGGGTCGCCGGAAA	TCAAAGACGACTGTCCACTCG
mSOX2	GCGGAGTGGAAACTTTTGTCC	CGGGAAGCGTGTACTTATCCTT
mKRT19	GGGGGTTCAGTACGCATTGG	GAGGACGAGGTCACGAAGC
mKRT7	AGGAGATCAACCGACGCAC	GTCTCGTGAAGGGTCTTGAGG
mSOX9	GAGCCGGATCTGAAGAGGGA	GCTTGACGTGTGGCTTGTTC
mAFP	CTTCCCTCATCCTCCTGCTAC	ACAAACTGGGTAAAGGTGATGG
mALB	TGCTTTTTCCAGGGGTGTGTT	TTACTTCCTGCACTAATTTGGCA
chipLSD1	TGCCAGGTCCCACTCCAAAC	ACAAAAAGGGTCGGAGACACC

Supplementary Table 5 Target sequences of shRNA and gRNA.

**Name**	Target sequence
mGDF1 gRNA-1	CGTCTTTGACCTGTCGAATG-TGG
mGDF1 gRNA-2	CCTGCGACACTACGAAGACA-TGG
GDF1 gRNA-1	TGTGGCGCCTGTTTCGACGC-CGG
GDF1 gRNA-2	CATCGCGCAGTCCTAGAGCC-TGG
ALK4-RNAi #1	CTGCTGCTACACTGACTACTG
ALK4-RNAi #2	AGCAGAGATATACCAGACGGT
ALK5-RNAi #1	GCGAGAACTATTGTGTTACAA
ALK5-RNAi #2	GCCTTGAGAGTAATGGCTAAA
ALK7-RNAi #1	CGGAGGAATTGTTGAGGAGTA
ALK7-RNAi #2	GCAACACCTCAACTCATCTTT
SMAD2-RNAi #1	CAGCAGAACTATCTCCTACTA
SMAD2-RNAi #2	AACCAGGAATTTGCTGCTCTT
SMAD3-RNAi #1	GCAACCTGAAGATCTTCAACA
SMAD3-RNAi #2	GCTTTGAGGCTGTCTACCAGT
