# Supplementary material for: Growth differentiation factor 1-induced tumour plasticity provides a therapeutic window for immunotherapy in hepatocellular carcinoma
Source: Nat Commun. 2021 Dec 8;12:7142. doi: 10.1038/s41467-021-27525-9 (PMC8654996; doi:10.1038/s41467-021-27525-9)
Supplement: Supplementary file 3 — Description of Additional Supplementary Files [file 41467_2021_27525_MOESM3_ESM.pdf]

Title: Supplementary Data 1:
Description: Gene list and expression in PCR array.

Title: Supplementary Data 2:
Description: Differentially expressed genes in RNA-seq data of PLC-8024-CTR and PLC-8024-GDF1 cells.

Title: Supplementary Data 3:
Description: List of CTA genes.
