# Supplementary material for: Growth differentiation factor 1-induced tumour plasticity provides a therapeutic window for immunotherapy in hepatocellular carcinoma
Source: Nat Commun. 2021 Dec 8;12:7142. doi: 10.1038/s41467-021-27525-9 (PMC8654996; doi:10.1038/s41467-021-27525-9)
Supplement: Supplementary file 7 — Reporting Summary [file 41467_2021_27525_MOESM7_ESM.pdf]

nature portfolio

Corresponding author(s): Ming Liu

Last updated by author(s): Nov 11, 2021

Reporting Summary

Nature Portfolio wishes to improve the reproducibility of the work that we publish. This form provides structure for consistency and transparency in reporting. For further information on Nature Portfolio policies, see our Editorial Policies and the Editorial Policy Checklist.

Statistics

For all statistical analyses, confirm that the following items are present in the figure legend, table legend, main text, or Methods section.

n/a	Confirmed	
[ ]	[x]	The exact sample size (*n*) for each experimental group/condition, given as a discrete number and unit of measurement
[ ]	[x]	A statement on whether measurements were taken from distinct samples or whether the same sample was measured repeatedly
[ ]	[x]	The statistical test(s) used AND whether they are one- or two-sided<br>*Only common tests should be described solely by name; describe more complex techniques in the Methods section.*
[ ]	[x]	A description of all covariates tested
[ ]	[x]	A description of any assumptions or corrections, such as tests of normality and adjustment for multiple comparisons
[ ]	[x]	A full description of the statistical parameters including central tendency (e.g. means) or other basic estimates (e.g. regression coefficient) AND variation (e.g. standard deviation) or associated estimates of uncertainty (e.g. confidence intervals)
[ ]	[x]	For null hypothesis testing, the test statistic (e.g. *F*, *t*, *r*) with confidence intervals, effect sizes, degrees of freedom and *P* value noted<br>*Give P values as exact values whenever suitable.*
[x]	[ ]	For Bayesian analysis, information on the choice of priors and Markov chain Monte Carlo settings
[x]	[ ]	For hierarchical and complex designs, identification of the appropriate level for tests and full reporting of outcomes
[ ]	[x]	Estimates of effect sizes (e.g. Cohen's *d*, Pearson's *r*), indicating how they were calculated

Our web collection on statistics for biologists contains articles on many of the points above.

Software and code

Policy information about availability of computer code

Data collection	Tumour formation and metastasis were evaluated with the IVIS Lumina XRMS Series III system (PerkinElmer, USA).
Data analysis	Graphpad Prism 9.0, SPSS 25, R 4.0.2 to perform statistical analyses.<br>Figures in this work were generated using Adobe Photoshop CC 2019.<br>RNA sequencing was performed at Shanghai Shenggong Biotech Co., Ltd. FastQC (version 0.11.2) was used for evaluating the quality of sequenced data. Raw reads were filtered by Trimmomatic (version 0.36). Highquality reads were aligned against the human genome assembly (National Center for Biotechnology Information build 37.1/hg19) using HISAT2 (version 2.1.0) with the RefSeq refGene annotation, which was downloaded from the University of California, Santa Cruz (UCSC) Genome Browser. RSeQC (version 2.6.1) was used for statistics of the alignment results and analysis of the redundancy sequences and the distribution of insert fragments. Qualimap (version 2.2.1) was used to check the homogeneity distribution and analysis of the genome structure. BEDTools (version 2.26.0) was used for statistical analysis of the gene coverage ratio and the distribution of sequenced sequence on chromosome. Transcripts abundances were quantified by StringTie (version 1.3.3b), which was used to calculate Transcripts Per Million (TPM) of both protein-coding genes and lncRNAs in each sample. For the samples without biological repetition, TMM was used to standardize the read count data, and then DEGseq (version 1.26.0)<br>was used for differential gene expression analysis.

For manuscripts utilizing custom algorithms or software that are central to the research but not yet described in published literature, software must be made available to editors and reviewers. We strongly encourage code deposition in a community repository (e.g. GitHub). See the Nature Portfolio guidelines for submitting code & software for further information.

Data

Policy information about availability of data

All manuscripts must include a data availability statement. This statement should provide the following information, where applicable:

- Accession codes, unique identifiers, or web links for publicly available datasets
- A description of any restrictions on data availability
- For clinical datasets or third party data, please ensure that the statement adheres to our policy

ENCODE: https://www.encodeproject.org. Human genome assembly GRCh37 (hg19): https://ftp.ncbi.nlm.nih.gov/genomes/all/annotation_releases/9606/105.20201022/GCF_000001405.25_GRCh37.p13/. The data in Supplementary Fig. 2e and Supplementary Fig. 4f used in this study are available in the Linkedomics web server database: http://www.linkedomics.org/login.php. The RNA-seq data generated in this study have been deposited in the GEO repository under accession code GSE188582. Raw data from each figure or table are provided as a Source Data file. Each single sheet of the Excel document was labeled with relevant figure or table. Uncropped images were provided as a separate sheet in the Excel file. Source data are provided with this paper.

Field-specific reporting

Please select the one below that is the best fit for your research. If you are not sure, read the appropriate sections before making your selection.

☒ Life sciences ☐ Behavioural & social sciences ☐ Ecological, evolutionary & environmental sciences

For a reference copy of the document with all sections, see nature.com/documents/nr-reporting-summary-flat.pdf

Life sciences study design

All studies must disclose on these points even when the disclosure is negative.

Sample size	A reasonable sample size was estimated from similar experiments in others' and our former publications (doi: 10.1126/scitranslmed.abb6282, doi: 10.1053/j.gastro.2019.01.252) to ensure adequate reproducibility of results. The exact n values used to calculate the statistics are provided.
Data exclusions	No data were excluded from the analyses.
Replication	All experiments were replicated independently. Number of repeats is provided in the text and figure legends where appropriate.
Randomization	Mice were randomly distributed and assigned to different treatment groups prior to the start of the treatment. For cell culture experiments, all treatments and controls were done together. Wells were randomly assigned for treatments.
Blinding	For cell culture experiments, investigators were not blinded to group allocation, but had no vested interest in the conclusion. The samples were processed simultaneously or in parallel in all experiments. For other experiments, such as genomics analysis, histological scoring, animal samples collection and image analysis were performed blinded.

Reporting for specific materials, systems and methods

We require information from authors about some types of materials, experimental systems and methods used in many studies. Here, indicate whether each material, system or method listed is relevant to your study. If you are not sure if a list item applies to your research, read the appropriate section before selecting a response.

Materials & experimental systems

n/a	Involved in the study	
☐	☒	Antibodies
☐	☒	Eukaryotic cell lines
☒	☐	Palaeontology and archaeology
☐	☒	Animals and other organisms
☐	☒	Human research participants
☒	☐	Clinical data
☒	☐	Dual use research of concern

Methods

n/a	Involved in the study	
☒	☐	ChIP-seq
☒	☐	Flow cytometry
☒	☐	MRI-based neuroimaging

Antibodies

Antibodies used	GDF1 (R&D, MAB6937, Monoclonal Mouse IgG2B Clone # 748450, Human, IF, 1:100), GDF1 antibody (Biorbyt, orb33665, Human, IHC,1:200, WB, 1;1000), GDF1 (Aviva system biology, OACD03794, Mouse, WB, 1:500, IHC, 1:400), GDF1 (abbexa, abx103259,

Mouse, IF, 10ug/ml, WB, 2ug/ml), CD8 (Abcam, ab93278, Human, IHC, 1/500), CD8 (Abcam, ab209775, Mouse, IHC, 1/2000), Granzyme B (Abcam, ab4059, Mouse, IHC, 1/100), Ki67 (R&D, AF7649, Mouse, IF, 1/100), Cytokeratin 19 (Abcam, ab52625, Human, IHC/IF, 1/500, Abcam, WB, 1:1000), SOX9 (Abcam, ab185966, Human, IHC/IF, 1/500, WB, 1:1000), AFP (Proteintech, 14550-1-AP, Human, IF, 1/100, IHC, 1:200, WB, 1/1000), AFP (Abcam, ab46799, Human, WB, 1/1000), EPCAM (Abcam, ab92469, Human, IHC/IF, 1/100), TTR (Abcam, ab75815, Human, IF, 1/100), HNF-4-alpha (Abcam, ab181604, Human, IHC, 1/2000, WB, 1/1000), GAGE12B/C/D/E (Aviva system biology, OAAB07499, Human, IHC, 1/100, WB, 1/1000), LSD1 (Abcam, ab129195, Human, WB, 1/10000), GAPDH (D4C6R) (Cell Signaling Technology, #51332, Human, Mouse, WB, 1/5000), Smad2/3 (D7G7) (Cell Signaling Technology, #8685, Human, WB, 1/1000, Chip,1/100), , Phospho-Smad2 (Ser465/467)/Smad3 (Ser423/425) (D27F4) (Cell Signaling Technology, #8828, Human, WB, 1/1000), SMAD2 (Abcam, ab40855, Human, WB, 1/10000), SMAD3 (Abcam, ab208182, Human, WB, 1/1000), Activin A Receptor Type IB/ALK4 (Abcam, ab109300, Human, WB, 1/10000), TGF-beta RI/ALK5 (R&D, AF3025, Human, WB, 1/1000), Activin Receptor Type 1C/ALK7 (Novus biologicals, NBP1-50659, Human, WB, 1/2000), Donkey Anti-Mouse IgG H&L (Alexa Fluor® 488) (Abcam, ab150105, 1:2000), Goat polyclonal Secondary Antibody to Mouse IgG - H&L (Alexa Fluor® 488) (Abcam, ab150113, 1:2000), Donkey polyclonal Secondary Antibody to Rabbit IgG - H&L (Alexa Fluor® 647) (Abcam, ab150075, 1:2000), Goat polyclonal Secondary Antibody to Rabbit IgG - H&L (Alexa Fluor® 594) (Abcam, ab150080, 1:2000), Donkey polyclonal Secondary Antibody to Sheep IgG - H&L (Alexa Fluor® 647)(Abcam, ab150179, 1:2000), Anti-rabbit IgG, HRP-linked Antibody (Cell Signaling Technology, #7074, 1:5000), Anti-mouse IgG, HRP-linked Antibody (Cell Signaling Technology, #7076, 1:5000), Recombinant Human GDF-1 Protein (R&D, 6937-GD), Recombinant Human TGF-beta 1 Protein (R&D, 240-B), InVivoPlus anti-mouse PD-1 (CD279) (Bio X Cell, RMP1-14, BP0146), InVivoPlus rat IgG2a isotype control (Bio X Cell, 2A3, BP0089).

Validation

GDF1 (R&D, MAB6937, https://www.rndsystems.com/cn/products/human-gdf-1-antibody-748450_mab6937).
GDF1 antibody (Biorbyt, orb33665, https://www.biorbyt.com/gdf1-antibody-orb33665.html).
GDF1 (Aviva system biology, OACD03794, https://www.avivasysbio.com/gdf1-antibody-oacd03794.html).
GDF1 (abbexa, abx103259, https://www.abbexa.com/gdf1-antibody-p-48386).
CD8 (Abcam, ab93278, https://www.abcam.cn/cd8-alpha-antibody-ep1150y-ab93278.html).
CD8 (Abcam, ab209775, https://www.abcam.cn/cd8-alpha-antibody-epr20305-ab209775.html).
Granzyme B (Abcam, ab4059, https://www.abcam.cn/granzyme-b-antibody-ab4059.html).
Ki67 (R&D, AF7649, https://www.rndsystems.com/cn/products/mouse-ki67-mki67-antibody_af7649).
Cytokeratin 19 (Abcam, ab52625, https://www.abcam.cn/cytokeratin-19-antibody-ep1580y-cytoskeleton-marker-ab52625.html).
SOX9 (Abcam, ab185966, https://www.abcam.cn/sox9-antibody-epr14335-78-ab185966.html).
AFP (Proteintech, 14550-1-AP, https://www.ptgcn.com/products/AFP-Antibody-14550-1-AP.htm).
AFP (Abcam, ab46799, https://www.abcam.cn/alpha-1-fetoprotein-antibody-ab46799.html).
EPCAM (Abcam, ab92469, https://www.abcam.cn/prealbumin-antibody-epr3219-ab92469.html).
TTR (Abcam, ab75815, https://www.abcam.cn/prealbumin-antibody-ep2929y-ab75815.html).
HNF-4-alpha (Abcam, ab181604, https://www.abcam.cn/hnf-4-alpha-antibody-epr16885-chip-grade-ab181604.html).
GAGE12B/C/D/E (Aviva system biology, OAAB07499, https://www.avivasysbio.com/gage12b-antibody-n-terminal-region-oaab07499.html).
GAPDH (D4C6R) (Cell Signaling Technology, #51332, https://www.cellsignal.cn/products/antibody-conjugates/gapdh-d4c6r-mouse-mab-hrp-conjugate/51332?site-search-type=Products&N=4294956287&Ntt=51332&fromPage=plp&_requestid=325308).
LSD1 (Abcam, ab129195, https://www.abcam.cn/kdm1lsd1-antibody-epr6825-nuclear-marker-and-chip-grade-ab129195.html)
Smad2/3 (D7G7) (Cell Signaling Technology, #8685, https://www.cellsignal.cn/products/primary-antibodies/smad2-3-d7g7-xp-rabbit-mab/8685?site-search-type=Products&N=4294956287&Ntt=smad2%2F3&fromPage=plp).
Phospho-Smad2 (Ser465/467)/Smad3 (Ser423/425) (D27F4) (Cell Signaling Technology, #8828, https://www.cellsignal.cn/products/primary-antibodies/phospho-smad2-ser465-467-smad3-ser423-425-d27f4-rabbit-mab/8828?site-search-type=Products&N=4294956287&Ntt=8828&fromPage=plp&_requestid=317790)
SMAD2 (Abcam, ab40855, https://www.abcam.cn/smad2-antibody-ep784y-ab40855.html).
SMAD3 (Abcam, ab208182, https://www.abcam.cn/smad3-antibody-epr19686-chip-grade-ab208182.html).
Activin A Receptor Type IB/ALK4 (Abcam, ab109300, https://www.abcam.cn/activin-a-receptor-type-ibalk-4-antibody-epr4815-ab109300.html).
TGF-beta RI/ALK5 (R&D, AF3025, https://www.rndsystems.com/cn/products/human-tgf-beta-ri-alk-5-antibody_af3025).
Activin Receptor Type 1C/ALK7 (Human, WB, 1/2000, Novus biologicals, NBP1-50659, https://www.novusbio.com/products/alk-7-activin-receptor-type-1c-antibody_nbp1-50659).
Donkey Anti-Mouse IgG H&L (Alexa Fluor® 488)(Abcam, ab150105, https://www.abcam.cn/donkey-mouse-igg-hl-alexa-fluor-488-ab150105.html).
Goat polyclonal Secondary Antibody to Mouse IgG - H&L (Alexa Fluor® 488) (Abcam, ab150113, https://www.abcam.cn/goat-mouse-igg-hl-alexa-fluor-488-ab150113.html).
Donkey polyclonal Secondary Antibody to Rabbit IgG - H&L (Alexa Fluor® 647) (Abcam, ab150075, https://www.abcam.cn/donkey-rabbit-igg-hl-alexa-fluor-647-ab150075.html).
Goat polyclonal Secondary Antibody to Rabbit IgG - H&L (Alexa Fluor® 594) (Abcam, ab150080, 1:2000, https://www.abcam.cn/goat-rabbit-igg-hl-alexa-fluor-594-ab150080.html).
Donkey polyclonal Secondary Antibody to Sheep IgG - H&L (Alexa Fluor® 647)(Abcam, ab150179, https://www.abcam.cn/donkey-sheep-igg-hl-alexa-fluor-647-ab150179.html).
Anti-rabbit IgG, HRP-linked Antibody #7074, https://www.cellsignal.cn/products/secondary-antibodies/anti-rabbit-igg-hrp-linked-antibody/7074?site-search-type=Products&N=4294956287&Ntt=hrp-linked+antibody&fromPage=plp).
Anti-mouse IgG, HRP-linked Antibody #7076, https://www.cellsignal.cn/products/secondary-antibodies/anti-mouse-igg-hrp-linked-antibody/7076?site-search-type=Products&N=4294956287&Ntt=hrp-linked+antibody&fromPage=plp).
Recombinant Human GDF-1 Protein (R&D, 6937-GD, https://www.rndsystems.com/cn/products/recombinant-human-gdf-1-protein_6937-gd).
Recombinant Human TGF-beta 1 Protein (R&D, 240-B, https://www.rndsystems.com/cn/products/recombinant-human-tgf-beta-1-protein_240-b).
InVivoPlus anti-mouse PD-1 (CD279) (Bio X Cell, RMP1-14, BP0146 https://d2a7cdyquyl45u.cloudfront.net/tds-sheets/BP0146-tds.pdf).
InVivoPlus rat IgG2a isotype control (Bio X Cell, 2A3, BP0089, https://d2a7cdyquyl45u.cloudfront.net/tds-sheets/BP0089-tds.pdf).

The antibodies used in the study are widely used and well validated in the literature according to the manufacturer's instructions.

Eukaryotic cell lines

Policy information about cell lines

Cell line source(s)	293FT as well as the HCC cell lines PLC-8024, Huh7, Hep3B, and HepG2 were purchased from ATCC, and HCC cell lines CLC1, CLC2, CLC5, CLC11, CLC13, and CLC16 were kind gifts by Prof. Li-Jian Hui from the Shanghai Institute of Life Science, Chinese Academy of Sciences.
Authentication	The cell lines except Hep3B and Hepal-6 were not authenticated. All cell lines were kept at low passages in order to maintain their identity. Cell morphology and behavior such as tumor growth and response to anti-PD1 therapy was consistent with expectations.<br>The cell lines Hep3B and Hepal-6 were authenticated by Cyagen Bioscience (Suzhou, China) and National Collection of Authenticated Cell Cultures (Shanghai, China). For cell line authentication of Hep3B and Hepal-6, DNA was extracted by a commercial kit fromAxygen. The 21-STRs including Amelogenin locus were amplified and separated on ABI 3730XL Genetic Analyzer. The signals were then analyzed by the software DSMZ tools.
Mycoplasma contamination	All cell lines were tested negative for mycoplasma contamination.
Commonly misidentified lines (See ICLAC register)	No commonly misidentified cell lines were used in this work.

Animals and other organisms

Policy information about studies involving animals; ARRIVE guidelines recommended for reporting animal research

Laboratory animals	BALB/c (male, 5-6weeks) and C57BL/6 (male, 5-6weeks) mice were purchased from Guangdong medical laboratory animal center (Guangzhou, China). All BALB/c and C57BL/6 mice were maintained under specific pathogen-free conditions at 22-26 oC with a 12:12 hour dark/light cycle and 40-70% humidity at Guangzhou Medical University.
Wild animals	No wild animals were employed in the study.
Field-collected samples	No field-collected samples were used in this study.
Ethics oversight	All animal experiments were approved by the review board of Guangzhou Medical University.

Note that full information on the approval of the study protocol must also be provided in the manuscript.

Human research participants

Policy information about studies involving human research participants

Population characteristics	Population characteristics of patients recruited into the study are described in Tables S2 of the manuscript.<br>Covariates in Table S2: demographic data (age, gender), clinical data (Child Pugh Score, tumor stage, histology of tumour (grade), tumour number, tumour size, etc) and biochemical data (AFP, ALT, AST, albumin, HBsAg).
Recruitment	HCC patients undergoing hepatectomy or liver transplantation at the Affiliated Cancer Hospital of Guangzhou Medical University and Sun Yat-Sen University Cancer Center were consecutively recruited to the study. HCC was diagnosed according to international guidelines, integrating history, physical examination, biochemistry, and imaging techniques obtained by multiphasic CT, and/or dynamic contrast-enhanced MRI. Diagnosis of HCC was further confirmed by histopathological examination of surgical resection specimens. All participants underwent a physical examination and had routine biochemical blood tests such as liver function tests, serological tests for hepatitis B surface antigen. Death date was obtained from clinical records.
Ethics oversight	HCC tissues used in the study were obtained from patients with HCC undergoing hepatectomy or liver transplantation at the Affiliated Cancer Hospital of Guangzhou Medical University and Sun Yat-Sen University Cancer Center, with informed consent obtained from all patients and protocol approved by the Institutional Review Board of Guangzhou Medical University and Sun Yat-Sen University. Samples were collected from patients who had not received any previous local or systemic treatment before operation.

Note that full information on the approval of the study protocol must also be provided in the manuscript.
